# Association between ageing population, median age, life expectancy and mortality in coronavirus disease (COVID-19)

**DOI:** 10.18632/aging.104193

**Published:** 2020-11-24

**Authors:** Xue-Qiang Wang, Ge Song, Zheng Yang, Ren-Jie Chen, Yi-Li Zheng, Hao-Yu Hu, Xuan Su, Pei-Jie Chen

**Affiliations:** 1 Department of Sport Rehabilitation, Shanghai University of Sport, Shanghai, China; 2 Department of Rehabilitation Medicine, Shanghai Shangti Orthopaedic Hospital, Shanghai, China; 3 School of Public Health, Key Lab of Public Health Safety of the Ministry of Education and NHC Key Lab of Health Technology Assessment, Fudan University, Shanghai, China

**Keywords:** mortality, coronavirus disease, COVID-19, median age, life expectancy

## Abstract

As of May 5, 2020, the number of confirmed coronavirus disease (COVID-19) cases has been more than 3.5 million with 243,540 deaths. We aimed to determine the associations between ageing population, median age, life expectancy at birth and COVID-19 mortality. The numbers of COVID-19 cases and deaths in the European region were obtained from the World Health Organization database. The data on percentage of the population aged 65 and over, median age and life expectancy at birth were extracted from the World Factbook of Central Intelligence Agency. A total of 56 countries/areas in the Europe reported COVID-19 cases and deaths (1,121,853 cases and 100,938 deaths) on April 20, 2020. The results showed significant positive associations between COVID-19 mortality and ageing population (r =0.274; P =0.021), median age (r =0.255; P=0.029), male median age (r =0.284; P =0.017), female median age (r =0.224; P=0.049), life expectancy at birth (r =0.336; P=0.006), male life expectancy at birth (r =0.342; P=0.005), female life expectancy at birth (r =0.312; P=0.01) in the 56 European countries/areas. This study illustrated that COVID-19 mortality was positively associated with ageing population, median age, and life expectancy at birth.

## INTRODUCTION

The World Health Organization (WHO) declared that the coronavirus disease (COVID-19) outbreak was characterized as a global pandemic on March 11, 2020 [1]. As of May 5, 2020, a total of 3,517,345 people infected with COVID-19 and 243,401 deaths in 215 countries/territories/areas were reported [2]. COVID-19 is still drastically spreading in the United States and Europe and the former had the greatest number of confirmed COVID-19 cases (61,906 deaths and 1,154,985 cases). The case fatality rates in France, the United Kingdom and Italy reached as high as 15.08% (28,734 deaths and 190,588 confirmed cases), 13.54% (15,464 deaths and 114,221 confirmed cases) and 13.72% (29,079 deaths and 211,938 confirmed cases), respectively [2].

As reported in previous studies [3–10], age was an important factor for the case fatality rate of COVID-19 patients. In the United States [7], COVID-19 patients aged ≥85 years (10% to 27%) had the highest case fatality rate, followed by aged 65-84 years (3% to 11%) and aged 20-54 years (<1%). In Italy [11], the reported case fatality rates were 22.7% for those aged ≥90 years, 19.7% for those aged 80-89 years, 12.5% for those aged 70-79 years and 3.5% for those aged 60-69 years. Furthermore, Wu C et al. [8] found that the old age (≥ 65 years) was associated with a greater risk of developing death (hazard ratio 6.17, 95% confidence interval 3.26-11.67). Similarly, another study [12] revealed that the old age (> 70 years) was significantly associated with the higher risk of death from COVID-19 (relative risk 10.67).

The case fatality rate of COVID-19 in Europe (9.29%) was higher than that in the Americas (5.39%), Western Pacific (4.09%), Eastern Mediterranean (3.8%), South-East Asia (3.69%) and African (3.41%) on May 5, 2020 [2]. Population age structure may explain the significant differences in case fatality rate across countries/territories/areas [13, 14]. In other words, the European region has the oldest populations among six WHO regions. The percentages of total population aged 65 years and over are estimated at 22.08% in Italy and 20.46% in France, compared with 16.85% in the United States and 12.34% in China [15]. In addition, the median ages are 46.5 years in Italy and 43.9 years in Spain, compared with 38.5 years in the United States and 38.4 years in China [15]. However, whether a relationship exists between population age structure and COVID-19 mortality in different countries/territories/areas remains unclear. Thus, this study was designed to determine the association between ageing population, median age, life expectancy at birth and COVID-19 mortality.

## RESULTS

The characteristics of COVID-19 mortality, ageing population, median age and life expectancy at birth in 56 European countries/areas were summarized in Table 1. As of April 20, 2020, a total of 56 countries/areas reported 100,938 deaths and 1,121,853 cases of COVID-19 in the European region. The median values of ageing population, median age, male median age, female median age, life expectancy at birth, male life expectancy at birth and female life expectancy at birth were 18.61%, 41.76 years, 40.15 years, 43.55 years, 80.2 years, 77.15 years and 82.85 years, respectively. The highest case fatality rate of COVID-19 came from France, at 17.43%. The ageing population was from 5.8% in Kyrgyzstan to 35.15% in Monaco. The highest median age and life expectancy at birth were 55.4 and 89.3 years, respectively, in Monaco.

**Table 1:** Dataset containing COVID-19 mortality, ageing population, median age, life expectancy in Europe region, as of April 20, 2020.

**Countries/areas**	**COVID-19 deaths (n)**	**COVID-19 cases (n)**	**COVID-19 mortality^a^ (%)**	**Ageing population^b^ in 2020 (%)**	**Median age in 2020 (years)**			**Life expectancy at birth in 2020 (years)**		
					**Total**	**Male**	**Female**	**Total**	**Male**	**Female**
Albania	26	548	4.74	13.03	34.3	32.9	35.7	79	76.3	81.9
Andorra	35	704	4.97	17.36	46.2	46.3	46.1	83	80.8	85.4
Armenia	20	1248	1.60	12.6	36.6	35.1	38.3	75.6	72.3	79.2
Austria	443	14662	3.02	19.87	44.5	43.1	45.8	81.9	79.2	84.7
Azerbaijan	18	1373	1.31	7.29	32.6	31.1	34.2	73.6	70.5	76.9
Belarus	45	4779	0.94	15.93	40.9	38	43.9	73.8	68.3	79.5
Belgium	5453	37183	14.67	19.21	41.6	40.4	42.8	81.4	78.8	84.2
Bosnia and Herzegovina	46	1268	3.63	16.22	43.3	41.6	44.8	77.5	74.5	80.7
Bulgaria	41	878	4.67	20.06	43.7	41.9	45.6	75	71.8	78.5
Croatia	39	1832	2.13	21.06	43.9	42	45.9	76.7	73.6	80.1
Cyprus	17	761	2.23	12.97	37.9	36.7	39.4	79.3	76.4	82.2
Czechia	181	6654	2.72	20.23	43.3	42	44.7	79.3	76.3	82.4
Denmark	346	7242	4.78	19.91	42	40.9	43.1	81.2	79.3	83.3
Estonia	38	1512	2.51	21	43.7	40.4	47	77.4	72.7	82.3
Finland	90	3681	2.44	22.26	42.8	41.3	44.4	81.3	78.4	84.4
France	19294	110721	17.43	20.46	41.7	40	43.4	82.2	79.1	85.4
Georgia	4	394	1.02	16.85	38.6	35.9	41.4	77	72.9	81.3
Germany	4294	139897	3.07	22.99	47.8	46.5	49.1	81.1	78.7	83.6
Greece	105	2207	4.76	22.43	45.3	43.7	46.8	81.1	78.5	83.8
Guernsey	9	236	3.81	20.23	44.3	43	45.6	82.8	80.1	85.7
Hungary	189	1916	9.86	20.69	43.6	41.5	45.5	76.7	73	80.6
Iceland	9	1760	0.51	15.47	37.1	36.6	37.7	83.3	81	85.6
Ireland	571	14758	3.87	13.82	37.8	37.4	38.2	81.2	78.9	83.7
Isle of Man	4	291	1.37	21.08	44.6	43.6	45.6	81.6	79.8	83.6
Israel	158	13107	1.21	11.96	30.4	29.8	31	83	81.1	85
Italy	23227	175925	13.20	22.08	46.5	45.4	47.5	82.5	79.8	85.3
Jersey	11	234	4.70	17.05	37.5	36	39.5	82.2	79.7	84.9
Kazakhstan	17	1546	1.10	8.43	31.6	30.3	32.8	72	66.8	76.8
Kosovo	12	510	2.35	7.75	30.5	30.2	30.8	72.7	70.5	75.1
Kyrgyzstan	5	554	0.90	5.8	27.3	26.1	28.5	71.8	67.7	76.2
Latvia	5	712	0.70	20.5	44.4	40.5	48	75.4	70.9	80.1
Liechtenstein	1	82	1.22	18.88	43.7	42	45.3	82.2	79.9	85
Lithuania	33	1298	2.54	20.45	44.5	40.2	48.2	75.5	70.3	81.1
Luxembourg	72	3537	2.04	15.37	39.5	38.9	40	82.6	80.1	85.2
Malta	3	426	0.70	21.3	42.3	41.2	43.5	82.8	80.7	85
Monaco	1	98	1.02	35.15	55.4	53.7	57	89.3	85.4	93.3
Montenegro	5	308	1.62	16.02	39.6	38.1	41.1	77.3	74.8	79.8
Netherlands	3601	31589	11.40	19.82	42.8	41.6	44	81.7	79.5	84.1
North Macedonia	49	1170	4.19	15.17	39	38	40	76.3	74.2	78.6
Norway	148	6984	2.12	17.43	39.5	38.8	40.2	82.1	80	84.4
Poland	347	8742	3.97	18.72	41.9	40.3	43.6	78.3	74.5	82.3
Portugal	687	19685	3.49	20.92	44.6	42.7	46.5	81.1	77.9	84.4
Republic of Moldova	60	2351	2.55	14.03	37.7	36.2	39.5	71.9	68	76
Romania	417	8418	4.95	17.58	42.5	41	44	76	72.6	79.7
Russian Federation	361	42853	0.84	15.53	40.3	37.5	43.2	71.9	66.3	77.8
San Marino	39	455	8.57	20.24	45.2	43.9	46.3	83.5	80.9	86.3
Serbia	117	5994	1.95	20	19.4	18.5	20.3	76.3	73.4	79.4
Slovakia	11	1089	1.01	17.05	41.1	39.6	42.7	77.8	74.3	81.6
Slovenia	70	1317	5.32	21.23	41.8	40.1	43.6	81.4	78.5	84.4
Spain	20043	191726	10.45	18.49	43.9	42.7	45.1	82	79	85.2
Sweden	1511	13822	10.93	20.59	41.1	40.1	42.1	82.4	80.4	84.5
Switzerland	1110	27322	4.06	18.73	42.7	41.7	43.7	82.8	80.5	85.3
The United Kingdom	15464	114221	13.54	18.48	40.6	39.6	41.7	81.1	78.8	83.5
Turkey	1890	82329	2.30	8.35	32.2	31.7	32.8	75.7	73.3	78.2
Ukraine	141	5449	2.59	17.03	41.2	38.2	44.3	72.9	68.2	77.9
Uzbekistan	5	1495	0.33	5.87	30.1	29.4	30.7	74.8	71.7	78

Abbreviations: COVID-19, coronavirus disease.

^a^ Mortality is case fatality rate of COVID-19, which is the number of confirmed deaths per the number of confirmed COVID-19 cases.

^b^ Ageing population is percentage of the population aged 65 and over in the total population.

**Figure 1:**
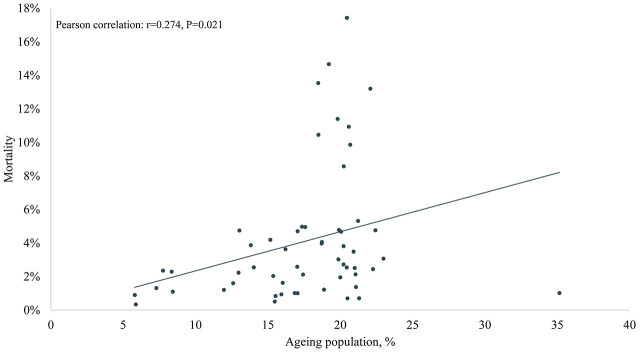
**The association of ageing population in 2020 and COVID-19 mortality in Europe.**

**Figure 2:**
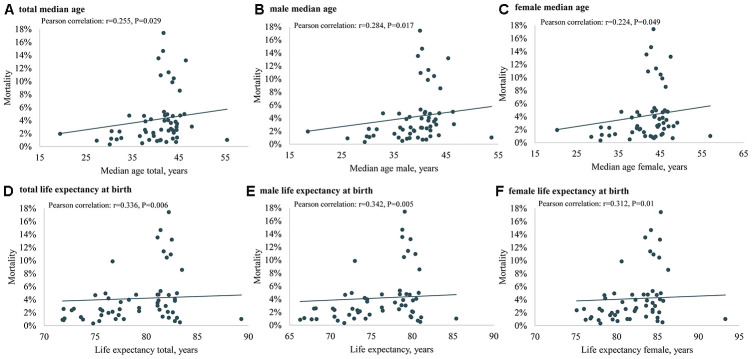
**The association of median age (total, male and female) in 2020, life expectancy at birth (total, male and female) in 2020 and COVID-19 mortality in Europe.** (**A**) total median age, (**B**) male median age, (**C**) female median age, (**D**) total life expectancy at birth, (**E**) male life expectancy at birth, (**F**) female life expectancy at birth.

The results of Pearson correlation analysis between ageing population, median age, life expectancy and COVID-19 mortality were shown in Figures 1 and 2. The results showed significant positive associations between COVID-19 mortality and ageing population (Pearson correlation: r =0.274, P=0.021), median age (Pearson correlation: r =0.255, P=0.029), male median age (Pearson correlation: r =0.284, P=0.017), female median age (Pearson correlation: r =0.224, P=0.049), life expectancy at birth (Pearson correlation: r =0.336, P=0.006), male life expectancy at birth (Pearson correlation: r =0.342, P=0.005), female life expectancy at birth (Pearson correlation: r =0.312, P=0.01).

**Figure 3:**
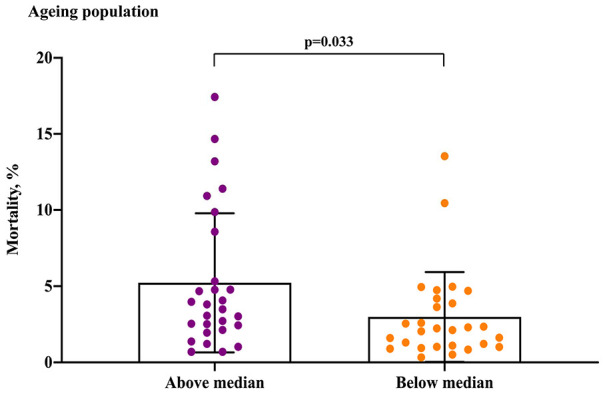
**COVID-19 mortality for ageing population in the above median group versus the below median group.**

**Figure 4:**
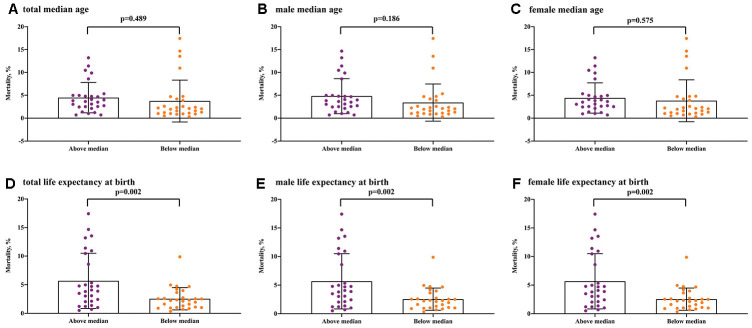
**COVID-19 mortality for median age and life expectancy at birth in the above median group versus the below median group.** (**A**) total median age, (**B**) male median age, (**C**) female median age, (**D**) total life expectancy at birth, (**E**) male life expectancy at birth, (**F**) female life expectancy at birth.

Regarding the ageing population in the 56 European countries/areas, COVID-19 mortality with 5.23% in the above median group was higher than 2.99% in the below median group (P= 0.033) (Figure 3). Regarding the median age, male median age and female median age, although the COVID-19 mortalities of the above median groups were slightly higher than the below median groups, there was no significant difference (median age, P=0.489; male median age, P= 0.186; female median age, P= 0.575) (Figure 4). Regarding the life expectancy, male life expectancy and female life expectancy at birth, the COVID-19 mortalities of the above median groups were significantly higher than the below median groups (P <0.01) (Figure 4).

## DISCUSSION

We estimated the association between ageing population, median age (total, male and female), life expectancy at birth (total, male and female) in 2020 and COVID-19 mortality in 56 European countries/areas. The results showed COVID-19 mortality was significantly associated with ageing population, median age (total, male and female) and life expectancy at birth (total, male and female).

Our study confirmed that a country with a greater percentage of the population aged 65 and over had higher COVID-19 mortality rate. In 56 European countries/areas, the mortalities in France (mortality 17.43% and 110,721 confirmed cases), the United Kingdom (mortality 13.54% and 114,221 confirmed cases) and Italy (mortality 13.23% and 175,925 confirmed cases) were higher than Turkey (mortality 2.3% and 82,329 confirmed cases) and Israel (mortality 1.21% and 13,107 confirmed cases) as of April 20, 2020. It could be explained by the reason that the percentages of the population aged 65 and over in France (20.46%), the United Kingdom (18.48%) and Italy (22.08%) are significantly higher than Turkey (8.35%) and Israel (11.96%). Moreover, we found the similar results between the European region and other regions. For instance, the mortalities in France, the United Kingdom, and Italy were higher than Brazil (mortality 6.4% and 36,599 confirmed cases) and Mexico (mortality 8.67% and 7,497 confirmed cases) as of April 20, 2020. These findings were consistent with previous studies [3–10] that demonstrated the older age (≥ 65 years) was a significant factor for the case fatality rate of COVID-19 patients. The main reason is that physiological disability with ageing, immune function decline and multimorbidity in older adults may lead to serious complications or even death. Furthermore, the hyperfunction theory of quasi-programmed aging explains why COVID-19 vulnerability (mortality) is an age-dependent syndrome and links it to other age-related diseases. Similarly, COVID-19 vulnerability (mortality) is associated with immunosenescence and cytokine storms as well [16]. Additionally, Dowd JB et al. [13] reported that diverse population age structure of different countries led to various mortality of COVID-19 around the world.

Furthermore, the case fatality rate of COVID-19 increased with median age across 56 countries/areas in this study. Chen T et al. [4] found that the median age of COVID-19 survivors (51 years) was significantly younger than that of non-survivors (68 years). Another study [8] reported that the median age of deceased COVID-19 cases (68.5 years) with acute respiratory distress syndrome was older than live patients (50 years). In this study, the COVID-19 mortality in Germany (mortality 3.07% and 139,897 confirmed cases) was obviously lower than Italy (mortality 13.23%, 175,925 confirmed cases), which could be explained by the relative lower median age of COVID-19 cases in Germany (48 years) and higher in Italy (64 years) [11, 13]. Similarly, the COVID-19 mortalities in France (17.43%) and Italy (13.2%) were higher than those in Israel (1.21%) and Uzbekistan (0.33%). The reason could be the median ages are 41.7 years in France and 46.5 years in Italy, compared with 30.4 years in Israel and 30.1 years in Uzbekistan. Meanwhile, there were the similar results between the European region and other regions. To illustrate, the COVID-19 mortalities of Span (10.45%), and Netherlands (11.4%) were higher than Argentina (mortality 4.65% and 2,839 confirmed cases) and Malaysia (mortality 1.65% and 5,389 confirmed cases) as of April 20, 2020. Correspondingly, the median ages are 43.9 years in Span and 41.6 years in Netherlands, compared with 32.4 years in Argentina and 29.2 years in Malaysia.

There were several major strengths in our study. First, to our knowledge, this work was the first to estimate the association between COVID-19 mortality with ageing population, median age, and life expectancy at birth in Europe. Second, since our study included 56 European countries/areas involving 100,938 deaths and 1,121,853 confirmed COVID-19 patients from the WHO databases, our results were reliably representative. Third, the subpopulation analyses (included total population, female and male) of median age and life expectancy at birth were conducted to further confirm the association between median age, life expectancy and COVID-19 mortality. These results further supported our hypothesis that COVID-19 mortality was positively correlated with median age and life expectancy at birth. Last but not least, our findings provided robust and novel evidence regarding the associations between ageing population and COVID-19 mortality in different countries/areas, which may explain the significant differences among COVID-19 mortality rates in the world.

This study had several limitations. First, although COVID-19 death was defined as “a death resulting from a clinically compatible illness in a probable or confirmed COVID-19 case, unless there is a clear alternative cause of death that cannot be related to COVID disease (e.g. trauma)” by the WHO [17], we could not eliminate the possibility of coding or diagnosis errors for death causes of COVID-19 in the 56 European countries/areas. In Italy, for example, a COVID-19 death is defined as a death occurring in a patient who has a positive laboratory-test result, while pre-existing diseases that may cause death are not considered [14]. Second, the ageing population, median age (total, male and female) and life expectancy at birth (total, male and female) of countries/areas in 2020 were provided and estimated by the World Factbook of Central Intelligence Agency [15]. Thus, this study may not be the true data of population age structure in the 56 European countries/areas. Third, despite our findings from Pearson correlation analyses that COVID-19 mortality was significantly associated with ageing population, median age and life expectancy at birth, COVID-19 mortality in a country/area was influenced by many factors, such as health-care workers, facilities, resources and public health policy for COVID-19 [18–20]. To explain further, Germany and Italy had similar ageing populations (22.99% for Germany and 22.08% for Italy), median age (47.8 years for Germany and 46.5 years for Italy) and life expectancy at birth (81.1 years for Germany and 82.5 years Italy) while significant differences existed in COVID-19 mortality between Germany (3.1%) and Italy (13.2%) as of April 20, 2020 [2]. This phenomenon could be caused by different public-health policy for COVID-19 (e.g., mandating isolation and banning gatherings), widespread testing for COVID-19 and healthcare resources (e.g., Germany had 29.2 critical care beds per 100,000 while Italy had 12.5) in Germany [21, 22]. We hoped that multiple linear regression analysis should be used to adjust for confounding factors. However, it was difficult for us to get the data, such as the number of health-care workers, physicians’ density, hospital beds, hospital beds density, medical care costs and other medical facilities from 56 countries/areas in the European region.

This study provides several implications for policy and practice during the COVID 19 pandemic. First, the older age is an important factor for the case fatality rate of COVID-19 patients. Our results suggest that old adults should protect themselves to avoid being infected with COVID-19 and reduce chances of spreading. However, significant differences exist in some simple precautions, such as regular hand washing, keeping at least 1 meter distance from others, avoiding crowded places, avoiding non-essential travel [23–25]. Second, comorbidity is another key risk of death from COVID-19. Therefore, elderly in comorbid conditions should be particularly vigilant and take protective measures to prevent COVID-19 infection. Old people with chronic diseases should maintain their medication supplies, take medications on time under the guidance of physicians and closely observe their symptoms of chronic diseases and disease progress. Third, growing numbers of older adults from 56 countries/areas in the European region and around the world are receiving care in long-term care facilities and nursing homes. Thus, all long-term care facilities and nursing homes should take protective measures to prevent the spread of COVID-19, including but not limited to restricting non-essential visitors, cancelling communal dining, monitoring fever and other symptoms of COVID-19 and providing necessary supplies (e.g. face mask) [26, 27]. Fourth, governments should provide additional financial supports, healthcare resources, public-health policy and support services for old adults during the COVID 19 pandemic.

## CONCLUSIONS

This study showed positive associations between COVID-19 mortality and ageing population, median age, and life expectancy at birth. Our findings can serve as a reference for old adults, medical staff, long-term care facilities and healthcare decision makers.

## MATERIALS AND METHODS

### Study population

This observational study was based on the WHO database on COVID-19 cases and deaths in European region as of 20 April 2020. All COVID-19 cases were confirmed through laboratory tests. A total of 56 countries/areas in the European region reported COVID-19 cases and deaths (1,121,853 cases and 100,938 deaths).

### Outcome measures

We included the following outcome measures: case fatality rate of COVID-19, ageing population, median age (total, male and female) and life expectancy (total, male and female) at birth. The case fatality rate of COVID-19 is the number of confirmed deaths per number of confirmed COVID-19 cases. The ageing population is the percentage of population aged 65 and over in the total population. According to the United Nations, a region with an ageing population of over 7% is regarded as an aging society, 14% as an aged society, and 20% as a super aged society [28]. Median age is the age that equally separates a population into two groups (half the population are older than median age and half are younger), which is considered as a key single index for the age distribution of a population. Life expectancy at birth, the mean number of years that a newborn may expect to live, is a summary measure for the mortality and health of a population [29, 30].

### Data sources

The numbers of COVID-19 cases and deaths in the European region were obtained from official situation reports issued by the WHO on April 20, 2020 [2]. We extracted the data on percentage of the population aged 65 and over, median age (total, male and female) and life expectancy (total, male and female) at birth in European countries/areas from the World Factbook of Central Intelligence Agency [15].

### Statistical analysis

We used SPSS 22.0 software (SPSS, Inc, Chicago, USA) to conduct statistical analyses in this study. Categorical and continuous variables were presented as n (%) and mean ± standard deviation (SD), respectively. For the primary analysis, Pearson correlation coefficients was used to determine the association between ageing population, median age (total, male and female), life expectancy at birth (total, male and female) in 2020 and COVID-19 mortality in Europe.

In additional secondary analyses, a median split was performed to divide the outcome measures into two groups: above and below the median value. To explain further, 56 European countries/areas were equally separated into above and below median groups according to ageing population (median = 18.61%), median age (median = 41.76 years), median male age (median = 40.15 years), median female age (median = 43.55 years), life expectancy at birth (median = 80.2 years), male life expectancy at birth (median = 77.15 years) and female life expectancy at birth (median = 82.85 years). We used independent sample t tests to analyse the significant difference in case fatality rate of COVID-19 between countries/areas above and below the median ageing population, median age and life expectancy at birth. Statistically significant differences would be determined with P values less than 0.05.
